# Nasal Septal Perforation Repair with an Inferior Turbinate Flap and Acellular Dermal Matrix

**DOI:** 10.1055/s-0040-1713418

**Published:** 2021-02-23

**Authors:** Saeid Mirzai, Andrew H. Lee, John J. Chi

**Affiliations:** 1 Alabama College of Osteopathic Medicine, Dothan, Alabama; 2 Department of Otolaryngology - Head & Neck Surgery, Johns Hopkins University School of Medicine, Baltimore, Maryland; 3 Division of Facial Plastic & Reconstructive Surgery, Department of Otolaryngology - Head & Neck Surgery, Washington University School of Medicine, St. Louis, Missouri

**Keywords:** nasal septal perforation, inferior turbinate flap, septal perforation repair, polydioxanone plate, acellular dermal matrix allograft

## Abstract

Nasal septal perforation is an uncommon disorder that can cause disturbance of nasal physiology. The perforations can vary widely in size, location, and symptomatology. Many different closure techniques have been described in the literature; however, no gold standard has been recognized. The choice of surgical technique usually depends on the characteristics of the perforation and surgeon experience. Due to the goal of perforation repair being restoration of normal nasal physiology, techniques with the best outcomes have been those resurfacing the septum with nasal respiratory mucosa. Here we present our novel surgical method for large (> 2 cm) septal perforation closure using a modification of the inferior turbinate flap repair using a polydioxanone plate and the acellular dermal matrix allograft (Alloderm, Allergan Inc.).

Nasal septal perforation has an estimated prevalence of 0.9 to 2.5%. 1 The most common cause is iatrogenic secondary to nasal surgery in addition to trauma, drug abuse, and autoimmune or idiopathic causes. 1 Treatment of septal perforations includes nasal irrigation, topical ointments, and septal silicon obturation. Many different surgical techniques have been described in the literature and success rates remain variable. 2 We present our novel surgical method for septal perforations > 2 cm using a modification of the inferior turbinate (IT) flap repair with a polydioxanone plate (PDS plate, Ethicon Inc.) and acellular dermal matrix (ADM) (Alloderm, Allergan Inc.).

## Materials and Methods

Between 2016 and 2018, five adult patients ( Table 1 ) underwent this novel surgical procedure. Adult patients with nasal septal perforations larger than 2 cm with postoperative follow-up of at least 3 months were included in the study. The study was approved by the Institutional Review Board.

**Table 1:** Patient demographics and characteristics

Sex	Preoperative symptoms	Follow-up (months)	Prior nasal surgery	History of nasal decongestant use	History of nasal trauma	Location	Size (cm)	Flap complication	Duration of splinting (weeks)	IT flap laterality
F	Obstruction, whistling, crusting, malodor	6	Yes, septoplasty	Yes	Yes	Anterior to mid-septum	2.5 × 1.5	None	4	Right
F	Obstruction, epistaxis, crusting, malodor	50	Yes, septoplasty	No	No	Anterior to mid-septum	2.5 × 2.0	None	4	Left
F	Obstruction	9	No	No	Yes	Anterior to mid-septum	2.5 × 1.0	None	4	Left
M	Obstruction, whistling, epistaxis, crusting, malodor	6	Yes, septoplasty	No	No	Anterior septum	2.5 × 1.5	None	4	Right
M	Obstruction, whistling, epistaxis, crusting, malodor	6	Yes, septoplasty	Yes	Yes	Anterior septum	2.5 × 1.5	None	4	Right

Abbreviations: F, female; IT, inferior turbinate; M, male.

## Results

Five patients (three women, two men) underwent successful repair of their nasal septal perforation using this novel surgical method. The mean age was 39.6 years (range: 22–56 years old). The median length of follow-up was 6 months (range: 6–50 months). No postoperative bleeding, revision surgeries, recurrent or residual perforations, empty nose syndrome, crusting, or other complications occurred. No complications at the IT donor site were noted. All of the IT donor sites remucosalized without issue. All of the patients reported an improvement in their preoperative symptoms (nasal obstruction, whistling, epistaxis, crusting, malodor).

## Surgical Technique

**Fig. 1:**
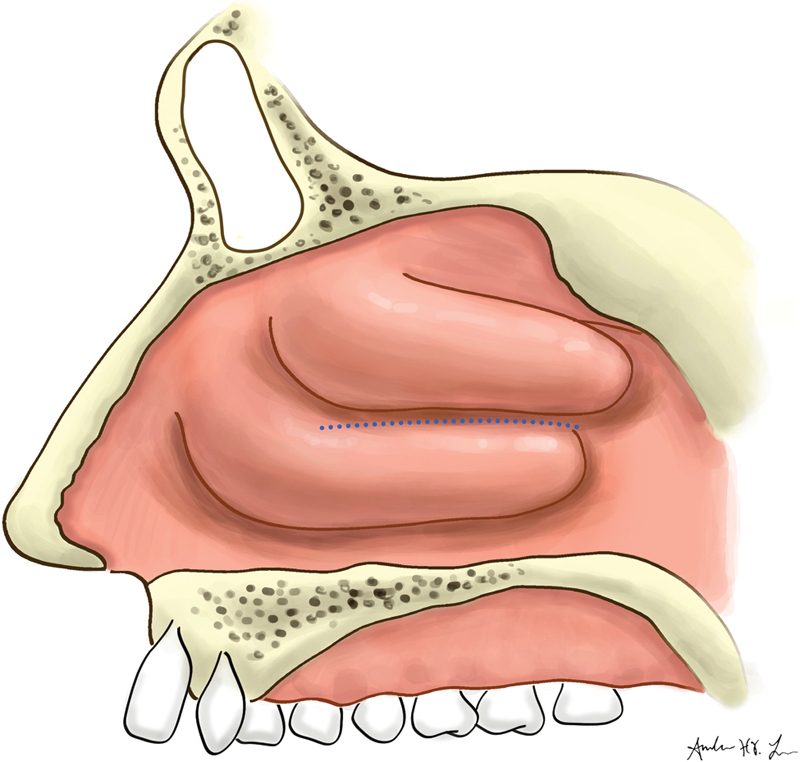
Line of separation of the inferior turbinate from the lateral nasal wall.

**Fig. 2:**
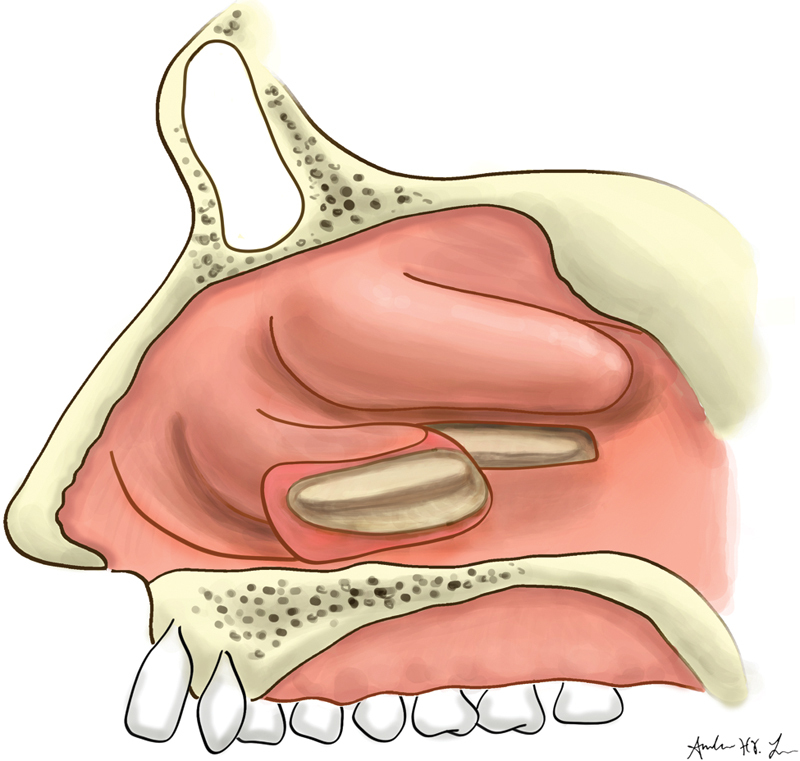
Separated inferior turbinate flap with 1- to 2-cm attachment to the lateral nasal wall.

This technique is a modification of IT flap harvest described by Murakami et al. 3 The flap is anteriorly based on the angular artery. An open septoplasty approach is used. Bilateral nasal septal flaps are elevated with great care around the perforation to minimize tearing of the flaps and further enlargement. After flap elevation, the IT flap is harvested. The laterality is determined by which side has a larger defect after nasal septal flap elevation. Ideally, the IT flap is elevated and harvested on that side. Using a zero-degree rigid nasal endoscope and a Cottle elevator, the IT is infractured, and back-biter forceps is used to separate the turbinate from the lateral nasal wall ( Fig. 1 ). A 2-cm attachment to the lateral nasal wall is preserved for vascularity ( Fig. 2 ). Next, the IT is rotated out of the nasal cavity, and the turbinate bone is removed.

Then a PDS plate is cut to size approximately 1 cm larger than the nasal septal perforation. A piece of ADM (0.33–0.76 mm thick) is secured to the PDS plate using a 5–0 PDS suture. This PDS plate/ADM complex is implanted between the nasal septal flaps, with the ADM oriented away from the side with the IT flap.

**Fig. 3:**
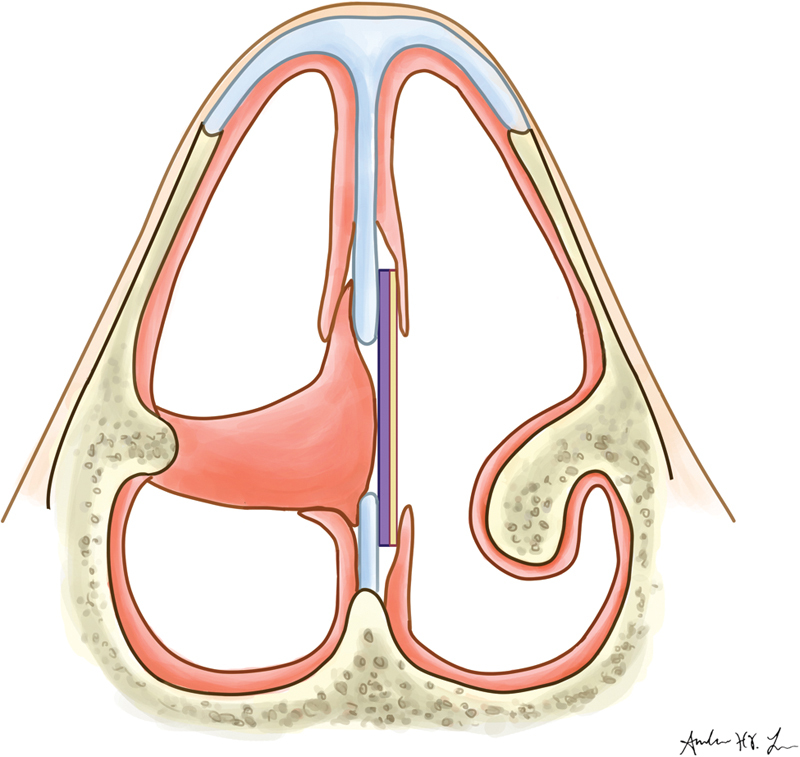
Final repair with inferior turbinate flap, polydioxanone plate (plate adjacent to flap), and acellular dermal matrix allograft (plate away from flap).

**Fig. 4:**
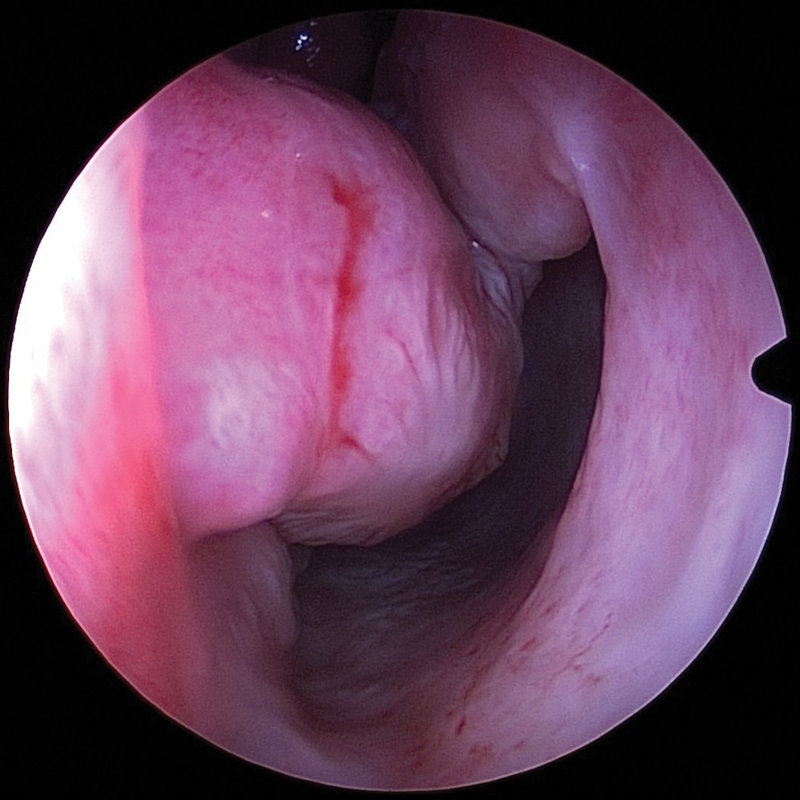
Predivision, preserved attachment of inferior turbinate to the lateral nasal wall at 6 to 8 weeks postoperative period.

**Fig. 5:**
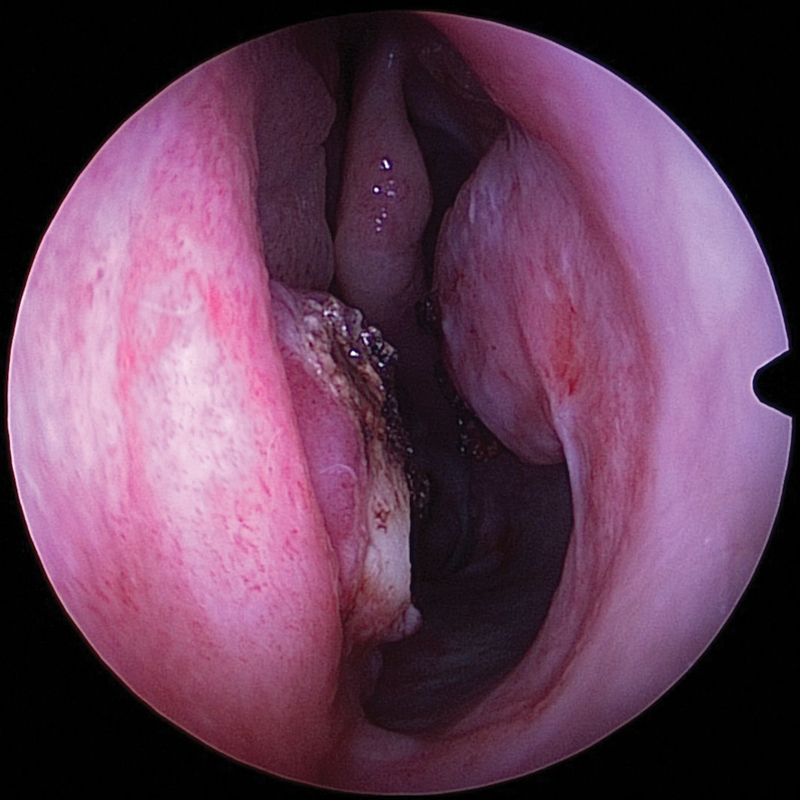
Postdivision flap used with a no. 15 blade, endoscopic scissors, and suction cautery for hemostasis.

Next, the IT flap is passed through the septal perforation, laid under the edges of the ipsilateral septal perforation, and secured to the contralateral nasal septal flap and the PDS plate/ADM complex using 3–0 chromic and 5–0 PDS sutures ( Fig. 3 ). A splint is placed in the nasal cavity contralateral to the IT flap for 2 to 4 weeks. The flap is divided 6 to 8 weeks later ( Fig. 4 ; Video 1 ) in the operating room. Under endoscopic guidance, the flap is divided with a scalpel and endoscopic scissors at the posterior limit of its attachment to the septal perforation ( Fig. 5 ; Video 2 ). A small approximately 1-cm portion of the IT flap pedicle is typically discarded to allow adequate separation between the septum and the IT stump to prevent synechia formation. The flap and the IT stump are then cauterized for hemostasis. No additional stents or packing is applied at this time.

## Discussion




**Video 1** Predivision, preserved attachment of inferior turbinate to lateral nasal wall at 6 to 8 weeks postoperative period.



**Video 2** Postdivision flap used with a no. 15 blade, endoscopic scissors, and suction cautery for hemostasis.



**Video 3** Predivision, healed side of interposition graft contralateral to the inferior turbinate flap.


The goal of nasal septal perforation repair is the restoration of normal nasal physiology such as laminar airflow, warming, humidification, and mucociliary function, which is accomplished by the techniques that restore nasal septal mucosa. 4 The perforation size impacts the repair and success of closure. Small perforations are treated successfully with local advancement flaps from remaining nasal septum and floor mucosa. 5 Perforations >2 cm are not easily repaired with local tissue. 4 Near-total and total perforations may require an extranasal flap, such as a pericranial flap, for closure. 1 These techniques supply a large amount of vascularized tissue but cause persistent dryness and crusting due to lack of mucociliary function. 4

The choice of repair technique and success rate is highly dependent on surgeon experience. Although there have been new techniques described in the literature, most surgeons have not adopted the more complex repairs, likely due to poor success rates, demonstrating the need for reproducible repair techniques. 4 The IT flap in our technique was first described by Murakami et al. The flap harvest has a robust anteriorly based blood supply from the angular artery. The flap has a wide arc of rotation, combined skeletal and epithelial support, and ease of harvest and insertion. It provides nasal respiratory mucosa to achieve normal nasal physiology. It is particularly useful in patients who have failed other attempts at closure. 5

**Fig. 6:**
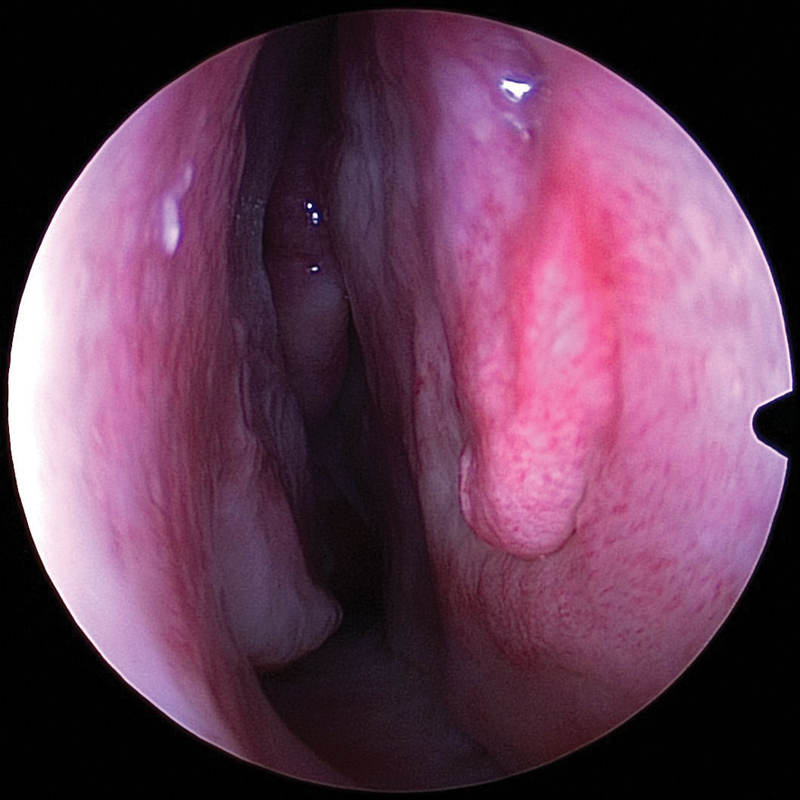
Predivision, healed side of the interposition graft contralateral to the inferior turbinate flap.

The major disadvantage of the IT flap is the need for a second procedure to divide the pedicle. The abundance of tissue that makes it a reliable flap can have enough bulk to cause partial nasal obstruction. 5 This is improved with the use of half of the turbinate, which preserves physiology and minimizes bulk. The flap is also limited by its unilateral coverage, where one side is not epithelialized and must heal by secondary ingrowth of epithelium. 5 In our modified technique, the use of interposition grafts promotes this epithelization and healing ( Fig. 6 ; Video 3 ). The ADM serves as a reservoir of growth factors that help promote collagen assembly and angiogenesis 4 and also serves as a bioabsorbable scaffolding for epithelial overgrowth. The PDS plate is readily available and easy to manipulate, and due to it lacking the potential for vascular and cellular ingrowth, 4 it is primarily used to implant and support the ADM on the nonflap side, minimizing the need for suturing the ADM to the septal flaps. The modified IT flap using a PDS plate and ADM is an effective, reliable, and novel treatment option for large (> 2 cm) septal perforations.

## References

[1] Santamaria-Gadea A Lopez-Chacon M Langdon C Modified nasal floor and inferior meatus flap for septal perforation repair. Extension and limits Rhinology 2018 56 04 386 392 30033453 10.4193/Rhin18.036

[2] Teymoortash A Hoch S Eivazi B Werner J A Experiences with a new surgical technique for closure of large perforations of the nasal septum in 55 patients Am J Rhinol Allergy 2011 25 03 193 197 21679532 10.2500/ajra.2011.25.3603

[3] Murakami C S Kriet J D Ierokomos A P Nasal reconstruction using the inferior turbinate mucosal flap Arch Facial Plast Surg 1999 1 02 97 100 10937085 10.1001/archfaci.1.2.97

[4] Delaney S W Kridel R WH Contemporary trends in the surgical management of nasal septal perforations: a community survey Facial Plast Surg 2019 35 01 78 84 30566987 10.1055/s-0038-1676049

[5] Friedman M Ibrahim H Ramakrishnan V Inferior turbinate flap for repair of nasal septal perforation Laryngoscope 2003 113 08 1425 1428 12897571 10.1097/00005537-200308000-00031

